# Interindividual differences contribute to variation in microbiota composition more than hormonal status: A prospective study

**DOI:** 10.3389/fendo.2023.1139056

**Published:** 2023-03-08

**Authors:** Zuzana Jackova, Jan J. Stepan, Stepan Coufal, Martin Kostovcik, Natalie Galanova, Zuzana Reiss, Karel Pavelka, Laszlo Wenchich, Hana Hruskova, Miloslav Kverka

**Affiliations:** ^1^ Laboratory of Cellular and Molecular Immunology, Institute of Microbiology, Czech Academy of Sciences, Prague, Czechia; ^2^ Institute of Rheumatology, Prague, Czechia; ^3^ Department of Rheumatology, First Faculty of Medicine, Charles University in Prague, Prague, Czechia; ^4^ Laboratory of Fungal Genetics and Metabolism, Institute of Microbiology, Czech Academy of Sciences, Prague, Czechia; ^5^ Department of Obstetrics and Gynecology, Charles University in Prague, First Faculty of Medicine, Prague, Czechia; ^6^ General University Hospital in Prague, Prague, Czechia

**Keywords:** microbiota, hormonal therapy, oophorectomy, bone metabolism, estrogen

## Abstract

**Aims:**

Ovarian hormone deficiency is one of the main risk factors for osteoporosis and bone fractures in women, and these risks can be mitigated by menopausal hormone therapy. Recent evidence suggests that gut microbiota may link changes in estrogen levels and bone metabolism. This study was conducted to investigate the potential relationship between hormonal and bone changes induced by oophorectomy and subsequent hormonal therapy and shifts in gut microbiota composition.

**Methods:**

We collected 159 stool and blood samples in several intervals from 58 women, who underwent bilateral oophorectomy. Changes in fecal microbiota were assessed in paired samples collected from each woman before and after oophorectomy or the start of hormone therapy. Bacterial composition was determined by sequencing the 16S rRNA gene on Illumina MiSeq. Blood levels of estradiol, FSH, biomarkers of bone metabolism, and indices of low-grade inflammation were measured using laboratory analytical systems and commercial ELISA. Areal bone mineral density (BMD) of the lumbar spine, proximal femur, and femur neck was measured using dual-energy X-ray absorptiometry.

**Results:**

We found no significant changes in gut microbiota composition 6 months after oophorectomy, despite major changes in hormone levels, BMD, and bone metabolism. A small decrease in bacterial diversity was apparent 18 months after surgery in taxonomy-aware metrics. Hormonal therapy after oophorectomy prevented bone loss but only marginally affected gut microbiota. There were no significant differences in β-diversity related to hormonal status, although several microbes (e.g., *Lactococcus lactis*) followed estrogen levels. Body mass index (BMI) was the most significantly associated with microbiota variance. Microbiota was not a suitable predictive factor for the state of bone metabolism.

**Conclusions:**

We conclude that neither the loss of estrogens due to oophorectomy nor their gain due to subsequent hormonal therapy is associated with a specific gut microbiota signature. Sources of variability in microbiota composition are more related to interindividual differences than hormonal status.

## 1 Introduction

Aging and postmenopausal decrease in the production of ovarian hormones are the main risk factors for accelerated bone loss, osteoporosis, and fractures in women (1). While 5-7 years of hormone therapy significantly reduces the risk of fractures (2), it is not enough to prevent postmenopausal osteoporosis (3). Estrogens act on osteoclasts, osteoblasts, and osteocytes (4, 5) in the bone microenvironment, mainly *via* their receptors (ERα, ERβ, and the G protein-coupled estrogen receptor). They increase the life span of osteoblasts and osteocytes by preventing their apoptosis (6) and may have an anabolic effect on the bone (7). Estrogen deficiency is associated with impaired osteoblast activity (8) and maturation of osteoclasts, decreased osteoclast apoptosis, and inflammatory immune responses (9–12). This effect is driven mainly by increased expression of pro-osteoclastogenic and inflammatory mediators by macrophages, dendritic cells, neutrophils, T cells, and B cells (10, 12) and increased reactive oxygen species formation and oxidative stress levels in the bone microenvironment (13). Consequently, estrogen deficiency results in the prevalence of osteoclastic bone resorption over bone formation, accelerated bone loss, and deteriorated bone quality (14, 15).

While different types of osteoporosis differ in their pathogenesis, the inflammatory response of innate (macrophages, dendritic cells, neutrophils) and adaptive immunity (B and T cells) always plays an important role (12, 16). Animal studies proposed several mechanisms of indirect regulation of bone mass by the microbiome. Expanding gut Th17 cells and subsequent pro-inflammatory tuning by segmented filamentous bacteria may lead to suboptimal skeletal development (17). Butyrate produced by clostridia in the gut leads to the expansion of regulatory T (Treg) cells that drive bone formation (18). Butyrate is also essential for the ability of the parathyroid hormone (PTH) to increase bone mass (19), again suggesting that hormonal regulation of bone metabolism may act through microbiota. This is especially important in sex steroid deficiency when gut permeability for luminal bacteria is increased. This in turn triggers inflammatory pathways that induce bone loss in conventional but not in germ-free mice (20). Translocated microbial products stimulate intestinal T cells that produce TNF and IL-17, which then move to the bone marrow, where they create an inflammatory environment and drive bone resorption (21). Apart from regulating gut permeability and inflammation, gut microbiota may regulate bone mineral density (BMD) *via* effects on calcium balance (transcellular and paracellular transport, intestinal lumen pH, short-chain fatty acids) (22), production of molecules with positive effects on the bone (23), and the effects on the endocrine system (IGF-1, serotonin, deconjugation of bile-secreted estrogens, biotransformation of polycyclic aromatic hydrocarbons into products with estrogenic activity) (24–26). The causal relationship between gut microbiota dysbiosis and impaired intestinal barrier function has also been demonstrated in a model of senile osteoporosis. The transfer of gut microbiota from osteoporotic rats to young rats impaired intestinal barrier function, increased bone turnover, and reduced bone mass in the recipients (27).

Only a few observational studies examined the associations between changes in estrogen levels, gut microbiome, and the quantity and quality of bone mass in humans, but their conclusions are not consistent. While some studies reported an increased relative frequency of Firmicutes and a decreased presence of Bacteroidetes in postmenopausal women compared with premenopausal women or (age-matched) men (28), others found similar microbiota composition in men and women and lower Firmicutes/Bacteroidetes ratio in women with high levels of estrogens (29, 30).

In summary, there are several possible mechanisms of association between the circulating estrogen levels and the actions of gut microbiota in postmenopausal osteoporosis. Under estrogen deficiency, the permeability of the intestinal wall increases (31), allowing bacterial products to enter the bloodstream. In response, the pro-inflammatory cytokines TNF-α and IFN-γ are released (32), further increasing gut permeability. This facilitates the passage of structural components and metabolic products of the intestinal microbiota (microbe-associated molecular patterns, short-chain fatty acids, endotoxins) into the circulation (33). Moreover, intestinal microbiota can migrate to the lamina propria, which again promotes inflammatory processes (34). Activated pro-inflammatory T cells can then migrate to the bone marrow, where they may influence bone remodeling (35). The aim of this study was to determine if acute changes in estrogen levels that lead to bone remodeling and bone mass decrease also induce marked gut dysbiosis in humans. The associations between changes in estrogens status, gut microbiome, BMD, and markers of bone remodeling and low-grade inflammation were prospectively examined in premenopausal women before and after undergoing oophorectomy, as well as in oophorectomized women on estrogen therapy.

## 2 Methods

### 2.1 Studied subjects

Between July 2018 and June 2020, out of 255 women who had bilateral oophorectomy, 160 women were invited for assessment of bone status through the Department of Obstetrics and Gynecology, General University Hospital in Prague. The exclusion criteria included estimated GFR <1.2 (ml/s/1.73 m^2^); alcohol abuse; diabetes mellitus; active neoplastic diseases; liver diseases; ascertained endocrine and rheumatologic diseases; immunosuppressive treatment; treatment with corticosteroids, aromatase inhibitors, anti-osteoporotic drugs, and anticonvulsants; and history of fragility fractures. Out of 56 eligible women who responded to our invitation, 13 women did not agree to follow-up visits, and 41 women agreed to baseline and follow-up dual-energy X-ray absorptiometry (DXA) and blood testing. Based on the gynecologist’s decision, hormone treatment was initiated in 16 women. The check-ups were performed 6 and 12 months after surgery or at the beginning of therapy.

Another 95 women were invited prior to a planned bilateral oophorectomy. Forty-two of these women agreed to baseline DXA and blood testing. Eleven were excluded: eight for receiving hormone therapy immediately after surgery and three for high biomarkers of bone remodeling. All patients were treated with ampicillin during surgery. In 31 women, DXA and blood testing were repeated 6 months after surgery, and hormone therapy was then initiated in nine of them, while 22 women were not treated. The check-ups were performed after 6 and 12 months. All women had BMD within ±1 T score range.

**Figure 1:**
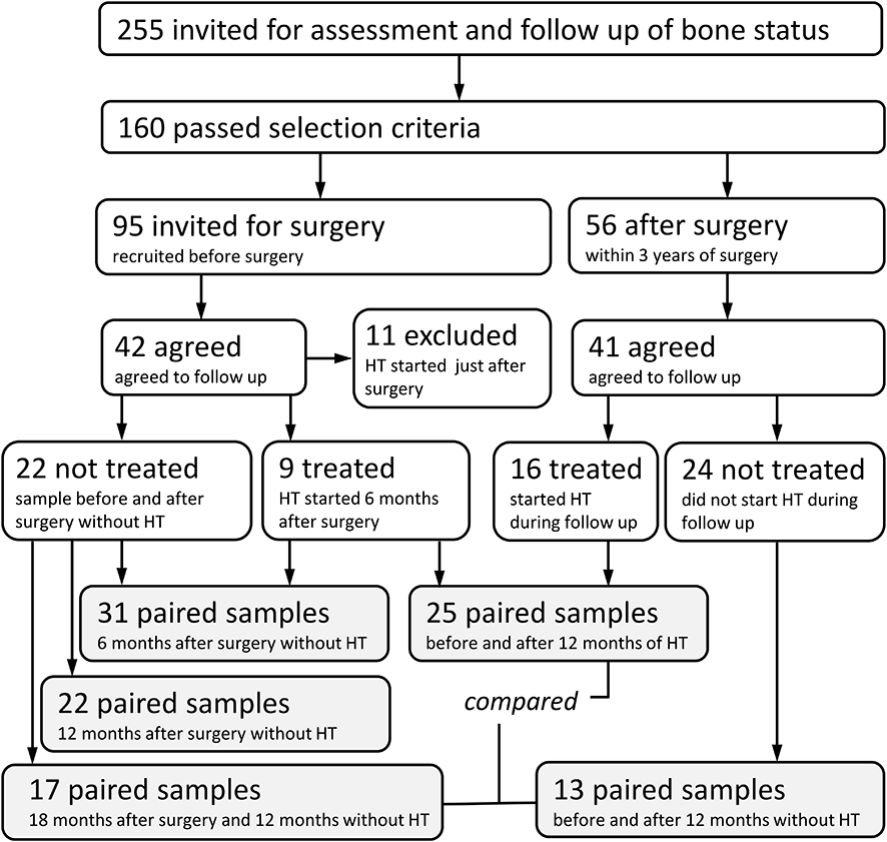
Study design.

Between July 2018 and August 2021, prospective evaluation was performed on 58 women scheduled for gynecological surgery or followed for post-surgery hormonal therapy and referred for bone evaluation by the Institute of Rheumatology, Prague, Czech Republic (**Figure 1**). The most common types of surgery that patients underwent were vaginal hysterectomy with adnexectomy performed laparoscopically (82.8% of 58 patients), bilateral adnexectomy (8.6%), abdominal hysterectomy with adnexectomy (3.4%), and adnexectomy post-abdominal hysterectomy (1.7%). The most common indications for surgery were benign tumors (58.6%), prevention or prophylaxis (e.g., BRCA mutation carriers, 19%), menstrual disorders and conditions related to menstrual bleeding (12.1%), and dysplasia and malignant tumors (5.2%). Patients were divided into three analyzed groups. The first group of 31 patients was sampled before and 6 months after the surgery. The second group of 25 patients was sampled after surgery, just before hormonal therapy, and after 12 months on estrogens. Additionally, we compared the fecal microbiota of the group on hormone therapy with 27 patients who were sampled after surgery and remained untreated for another 12 months. The active substance used for hormonal therapy was Estradiolum hemihydricum (Estrofem, 1 mg daily) in 22 patients and tibolonum (Ladybon, 2.5 mg daily) in two patients. No subject had bone or calcium metabolic disease or was receiving drugs that are known to affect bone or calcium metabolism. No woman had received hormone replacement therapy before the study. The subjects were advised to maintain their usual physical activity and diet throughout the study.

### 2.2 Ethical approval

This study was approved by the Ethics Committee of the Institute of Rheumatology, Prague, Czech Republic (Ref. number 5483/2017), and written informed consent was obtained from all participants before their enrolment. All study procedures were performed in compliance with the laws and regulations governing the use of human subjects (Declaration of Helsinki).

### 2.3 Sample collection and processing

During the baseline medical examination, the medical history of each patient was recorded. Clinical and laboratory parameters were then measured usually every 6 months together with stool and blood sample collection. Stool samples were freshly collected in standardized, sterile collection tubes by the participants and brought for their routine visits. All stool samples were delivered within 4 h of collection and immediately frozen at −80°C until analysis for microbiota composition. Venous blood samples were obtained after an overnight fast, centrifuged at 1,200×*g* for 15 min at 4°C, and serum or EDTA plasma samples were divided into aliquots and stored at −80°C until analysis.

### 2.4 Bone densitometry

Areal BMD of the lumbar spine, proximal femur, and femur neck was evaluated using DXA (GE Healthcare Lunar software version 14.1). Results were expressed in g/cm^2^ and the T-score was calculated using the National Health and Nutrition Examination Survey (NHANES) as reference. Quality control assurance measurements were performed in accordance with the manufacturer’s recommendations. The short-term *in-vivo* precision error for the lumbar spine (L1–L4), total femur, and femur neck was 0.7%, 0.9%, and 1.8%, respectively, and the long-term *in-vivo* precision error was 0.31%. Trained examiners with long-time experience conducted the measurements.

### 2.5 Biochemical analysis

The concentrations of serum total bone alkaline phosphatase (B-ALP), 25-hydroxyvitamin D [25(OH)D], and PTH (Cat. Nos. 310970, 310600, and 310630, respectively) were determined using the Liaison XL (Diaorin S.p.A., Saluggia, Italy) analytical system. Beta-carboxy-terminal type I collagen crosslinks (β-CTX), procollagen 1 N-terminal propeptide (P1NP), estradiol, follicle-stimulating hormone (FSH), and thyroid-stimulating hormone (TSH) (Cat. Nos. 09005773190, 03141071190, 06656021190, 11775863122, and 08429324190, respectively) were determined using the Cobas e601 (Roche, Basel, Switzerland) analytical system. Serum hsCRP, creatinine, glucose, phosphate, calcium, and γ-glutamyltransferase (GMT) (Cat. Nos. OSR6199, OSR61204, OSR6121, OSR6122, OSR61117, and OSR6120, respectively) were determined using the Beckman Coulter AU680 (Beckman Coulter, USA) analytical system. Serum IL-6 receptor (IL-6R) was quantified using the Human IL-6R alpha Quantikine ELISA (Cat. No. DR600, Bio-Techne, Minneapolis, MN, USA), EDTA plasma tumor necrosis factor receptor 1A (TNF-R) was determined using the sTNF-R (60 kDa) Human ELISA (Cat. No. RAF114R, Biovendor, Brno, Czech Republic), and EDTA plasma sclerostin was assessed using the Bioactive Sclerostin ELISA (Cat. No. BI-20472, Biomedica Medizinprodukte GmbH, Vienna, Austria).

### 2.6 DNA isolation and sequencing

The bacterial composition in stool samples was analyzed by 16S rRNA amplicon-based Illumina MiSeq sequencing as previously published, with minor modifications (36). Briefly, bacterial DNA from stool samples was extracted using ZymoBIOMICS™ DNA Miniprep Kit (Zymo Research, Irvine, CA, USA), according to the manufacturer’s protocol. The extracted DNA was measured by NanoDrop (Thermo Fisher Scientific, Waltham, USA) and diluted to 10 ng/µl. Next, the V3-V4 region of the bacterial 16S rRNA gene was amplified by PCR with degenerate primers 341F (5′-CCTACGGGNGGCWGCAG-3′) and 806R (5′-GGACTACHVGGGTWTCTAAT-3′). Each 25-µl PCR reaction contained either 5 µl of template or UltraPure™ DNase/RNase-Free distilled water (Thermo Fisher Scientific) as non-template control. The amplification was performed with KAPA HiFi HotStart Ready Mix (Roche), containing 12.5 µl of 2× KAPA HiFi HotStart ReadyMix, 0.75 µl of 10 μM forward primer, 0.75 µl of 10 μM reverse primer, and 6 µl of distilled water. Thermal cycling parameters were 35 cycles of denaturation (95°C, 3 min), annealing (60°C, 30 s), extension (72°C, 30 s), and final elongation (72°C, 5 min). The length of the amplicons was verified on agarose gel electrophoresis, and three PCR products from each sample were pooled to minimize random PCR bias. Next, these pooled PCR amplicons were normalized by the SequalPrep™ Normalization Plate (96) Kit (Thermo Fisher Scientific). The whole library was then concentrated to 50 µl in a centrifugal vacuum concentrator (Eppendorf SE, Hamburg, Germany) and ligated to sequencing adapters with the KAPA HyperPrep Kit (Roche). Next, the DNA concentration was quantified with the KAPA Library Quantification kit (Roche) and used for amplicon sequencing on the MiSeq platform (Illumina, San Diego, CA, USA) at the CEITEC Genomics Core Facility (Brno, Czech Republic).

### 2.7 Bioinformatics

Sequencing data were processed using QIIME 2.0 2021.8 (37). Raw reads were first demultiplexed and quality filtered using the q2-demux plugin and then denoised using the DADA2 algorithm, and a feature table with counts of amplicon sequence variants (ASVs) per sample was produced (38). Taxonomy was assigned using the q2-feature-classifier classify-sklearn (39), using a trained naive Bayes classifier against the SILVA_138_SSURef_Nr99 bacterial reference database. Rarefaction analysis of the final ASV tables was performed to assess the completeness of the dataset and the admissible data resampling level for statistical analysis. Both α-diversity (within samples) and β-diversity (among samples) were calculated with QIIME 2.0.

Faith’s phylogenetic diversity (PD), observed ASVs, Shannon diversity index, Simpson’s evenness measure *E*, and Pielou’s evenness index were analyzed as different metrics of α-diversity. Faith’s PD reflects community diversity with incorporated information on phylogenetic relationships, the Shannon diversity index indicates community diversity without phylogenetic relationships, and observed ASVs reflect community richness. Variation in α-diversity was analyzed using the linear mixed-effects (LME) models in a longitudinal manner with α-diversity indices as the response variable and patient identity defining the dependent samples, to account for repeated-measures sampling experiment design.

Beta diversity was presented in principal coordinate analysis (PCoA) plots and assessed using weighted (quantitative) UniFrac distances. Permutational multivariate analysis of variance (PERMANOVA) was used to confirm the statistical significance of the differences in β-diversity between patient groups. Adonis (permutational multivariate analysis of variance using distance matrices) from the treated/untreated groups based on weighted UniFrac dissimilarity was performed to investigate and rank the effect of nine variables commonly associated with variation in overall microbial composition (40, 41).

Changes in microbial abundances after intervention (surgery or hormonal therapy) were ranked with Songbird analysis (42) while accounting for several covariates. Only the models that converged under the baseline model and with the highest predictive accuracy (highest pseudo *Q* ^2^) are shown. The statistical significance of the differences in microbiota composition was analyzed by differential abundance analysis (ANCOM) (43). Sequences are publicly available in the Sequence Read Archive (SRA) under the BioProject accession number PRJNA914622.

### 2.8 Statistics

All variables were tested for normal distribution by the D’Agostino–Pearson test. Based on the results, the paired data were compared using a non-parametric Wilcoxon matched-pairs signed rank test or a paired *t*-test. Differences in continuous variables between two independent groups were analyzed by a non-parametric Mann–Whitney test or by an unpaired *t*-test. Comparison of multiple experimental groups was performed using a non-parametric Kruskal–Wallis or Friedman test with Dunn’s multiple comparison test or by two-way ANOVA with Sidak’s multiple comparison test. Statistical comparison was performed with GraphPad Prism statistical software (version 8.0.2., GraphPad Software, San Diego, CA, USA).

## 3 Results

### 3.1 Oophorectomy markedly changes the biomarkers of bone metabolism but not gut microbiota composition

**Figure 2:**
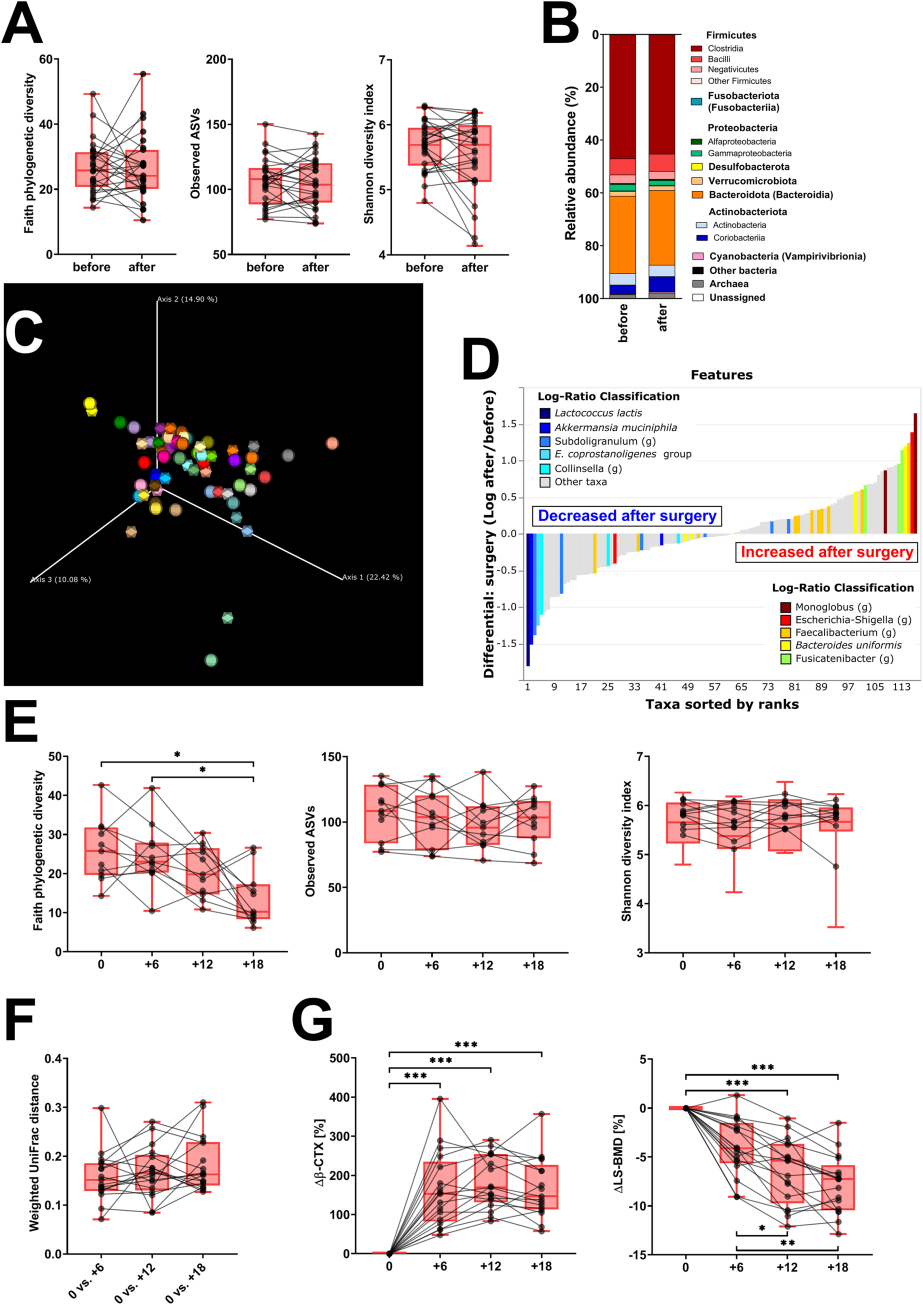
Fecal bacteria composition is not markedly changed by surgery. **(A)** Changes in α-diversity after surgery were characterized by the metrics Faith’s phylogenetic diversity, observed features, and Shannon diversity index. **(B)** β-Diversity comparison at the class level. **(C)** Principal coordinate analysis (PCoA) plot using the weighted UniFrac distance metric shows the fecal microbiota compositional differences induced by surgery. The time point before surgery is shown as a sphere and the time point after surgery as an icosahedron, and the same color represents the same patient. **(D)** Differential rankings of taxa associated with the patient’s status related to surgery with ranks estimated from multinomial regression with the top 5 species/genera related to each state highlighted. **(E)** Changes in α-diversity 6, 12, and 18 months after surgery; **(F)** changes in weighted UniFrac distances from the time before surgery; and **(G)** serum change in β-CTX (Δβ-CTX) and lumbar spine density (ΔLS-BMD) in the same time points. For α-diversity analyses, rarefaction was performed at 2,020 reads, and patients with lower yields in one time point were excluded from the analysis. Groups were compared with the Wilcoxon matched-pairs signed rank test or paired *t*-test **(A)** or Friedman test with Dunn’s multiple comparison test **(E–G)**. *p < 0.05; **p < 0.01; ***p < 0.001. Comparison of all clinical data for these patients is found in **Table 1**.

We evaluated the samples from 31 patients sampled before oophorectomy and 6 months later. Oophorectomy significantly decreased BMD and serum estradiol levels, while it increased the levels of FSH and markers of bone remodeling and low-grade inflammation (**Table 1**). No significant changes were found for hsCRP, creatinine, GMT, and TSH (data not shown). On the other hand, surgery did not induce significant changes in α-diversity (**Figure 2A**; **Supplementary Figure 1A**) or β-diversity (**Figures 2B, C**), despite the mild trend of decreased α-diversity. The changes in microbial abundance were ranked with Songbird, testing the effect of surgery with BMI and GMT included as random effects. Although these variables improved the model, its predictive accuracy on cross-validation samples was low (pseudo *Q* ^2^ = 0.06961). By ranking the identified microbes, this analysis allows for the identification of the strongest microbial feature associated with a particular variable. Changes in the abundance of several taxa were associated with the state after the surgery, although the associations were not very strong and several taxa were defined with multiple sequence variants associated even with the opposite state (**Figure 2D**). Therefore, while certain microbes have higher (e.g., genus *Faecalibacterium* or *Bacteroides uniformis*) or lower (e.g., *Lactococcus lactis* or *Akkermansia muciniphila*) abundance after surgery, the differences induced by surgery were quite low. In a subset of patients that were followed for a longer time, there was a significant decrease in Faith’s PD 18 months after surgery as well as a small decrease in Pielou’s evenness 6 months after surgery, while other metrics of α-diversity and overall shifts in β-diversity remained the same (**Figures 2E, F**; **Supplementary Figure 1B**). In this long-followed cohort, the increase in serum β-CTX and P1NP reached maximum levels 6 and 12 months after surgery, respectively. The values of markers of bone remodeling, sclerostin, TNF-R, IL-6R, and FSH remained increased over 6 to 12 months. Consequently, the decrease in spine and femur BMD continued progressively (**Table 1**; **Figure 2G**).

**Table 1:** Prospective laboratory data in women before and after oophorectomy.

Variable	Before	6 months	12 months	18 months	*p*
Follow-up (years)	0	0.55 ± 0.11	1.07 ± 0.13	1.55 ± 0.18	
*n*	31	31	22	11	
Age (years)	47.6 ± 4.3				
BMI (kg/m^2^)	27.9 ± 6.0	28.1 ± 6.5	28.4 ± 6.6	28.4 ± 6.3	0.157
Spine BMD (g/cm^2^)	1.272 ± 0.157	1.216 ± 0.150^*^	1.189 ± 0.144^*,**^	1.146 ± 0.130^*,**^	<0.001
ΔSpine BMD (%)	0	−4.42 ± 2.72^*^	−6.46 ± 2.98^*,**^	−7.81 ± 2.98^*,**^	<0.001
Proximal femur BMD (g/cm^2^)	1.079 ± 0.171	1.052 ± 0.164	1.023 ± 0.166^*,**^	0.990 ± 0.151^*,**^	<0.001
ΔProximal femur BMD (%)	0	−2.42 ± 2.64^*^	−3.66 ± 3.18^*^	−3.94 ± 3.67^*,**^	<0.001
Femur neck BMD (g/cm^2^)	1.010 ± 0.159	0.984 ± 0.151^*^	0.979 ± 0.168^*^	0.937 ± 0.157^*^	<0.001
ΔFemur neck BMD (%)	0	−2.45 ± 3.29^*^	−3.14 ± 3.51^*^	−4.07 ± 4.31^*^	<0.001
Glucose (mmol/L)	5.53 ± 0.76	5.66 ± 1.03	5.97 ± 1.24	6.12 ± 1.89	0.007
Phosphate (mmol/L)	1.08 ± 0.14	1.28 ± 0.14^*^	1.28 ± 0.12^*^	1.25 ± 0.12^*^	<0.001
Calcium (mmol/L)	2.38 ± 0.10	2.49 ± 0.11^*^	2.46 ± 0.08^*^	2.45 ± 0.08^*^	<0.001
PTH (pmol/L)	3.03 ± 1.18	2.54 ± 1.16	2.57 ± 1.11	3.17 ± 2.00	0.017
25(OH)D (nmol/L)	51.1 ± 21.3	65.9 ± 22.7^*^	71.4 ± 19.3^*^	67.9 ± 22.2^*^	0.002
β-CTX (μg/L)	0.26 ± 0.10	0.64 ± 0.27^*^	0.70 ± 0.25^*^	0.67 ± 0.20^*^	<0.001
Δβ-CTX (%)	0	161.2 ± 86^*^	185.6 ± 71^*^	167.8. ± 77^*^	<0.001
P1NP (μg/L)	44.6 ± 15.6	75.9 ± 30.4^*^	93.4 ± 34.7^*,**^	85.7 ± 29.2^*^	<0.001
ΔP1NP (%)	0	77.9 ± 72.0^*^	117.1 ± 88.5^*,**^	97.4 ± 77.2^*^	<0.001
Osteocalcin (μg/L)	17.3 ± 5.7	26.3 ± 10.0^*^	30.7 ± 10.4^*^	31.1 ± 11.4^*^	<0.001
B-ALP (μg/L)	9.6 ± 4.1	14.1 ± 6.1^*^	17.2 ± 7.2^*,**^	17.7 ± 8.7^*,**^	<0.001
Sclerostin (pmol/L)	105.4 ± 35.9	129.1 ± 39.1^*^	115.2 ± 36.0^*,**^	108.0 ± 22.1^*^	<0.001
ΔSclerostin (%)	0	25.4 ± 18.3^*^	14.3 ± 14.4^*,**^	16.9 ± 22.7^*,**^	<0.001
TNF-R (µg/L)	1.65 ± 0.69	2.02 ± 0.83^*^	1.86 ± 0.78^*^	1.46 ± 0.51^*^	<0.001
ΔTNF-R (%)	0	25.9 ± 36.1^*^	17.7 ± 33.8^*^	12.8 ± 39.4	<0.001
IL-6R (µg/L)	39.7 ± 9.4	44.1 ± 11.1^*^	44.1 ± 11.0^*^	40.9 ± 7.8	<0.001
ΔIL-6R (%)	0	11.7 ± 14.7^*^	10.8 ± 16.8^*^	3.2 ± 11.5	<0.001
Estradiol (pmol/L)	458.5 ± 315.8	35.4 ± 23.5^*^	39.2 ± 34.1^*^	42.9 ± 33.3^*^	<0.001
FSH (IU/L)	11.1 ± 11.2	82.7 ± 23.9^*^	76.9 ± 29.2^*^	82.7 ± 30.6^*^	<0.001

Data are mean (SD). Multiple comparisons using the one-way repeated measures ANOVA with Bonferroni t-test. No significant changes were found for hsCRP, creatinine, GMT, and TSH (data not shown). The full dataset is in the **Supplementary Table 1**.

^*^p < 0.05 vs. before; ^**^p < 0.05 vs. 6 months.

### 3.2 Hormonal therapy after oophorectomy has only a marginal effect on gut microbiota

Fifty-two women were examined approximately 1 year after oophorectomy and followed up for 1 year. Of these women, 27 received menopausal hormone therapy shortly after the initial examination and 25 were left untreated. As compared with baseline measurement, biomarkers of bone remodeling, sclerostin, TNF-R, IL-6R, and FSH remained significantly increased in untreated women, while their lumbar spine BMD decreased (**Table 2**). Treated women had a significant increase in BMD at the lumbar spine and femur neck, an increase in serum estradiol, and a significant decrease in serum FSH, calcium, phosphate, β-CTX, P1NP, osteocalcin, bone ALP, sclerostin, IL6-R, and plasma TNF-R (**Table 2**).

**Table 2:** Comparison of laboratory data in treated and untreated oophorectomized women.

Variable	0	Untreated	Treated	*p*
*n*	52	27	25	
Age	50.0 (46.5; 51.8)	50.5 (46.6; 52.0)	52.2 (47.8; 53.3)	0.220
Years since oophorectomy	0.89 (0.45; 1.55)	0.64 (0.43; 1.27)	1.04 (0.48; 1.67)	0.462
Follow-up (years)		1.00 ± 0.15	1.08 ± 0.12	0.061
BMI (kg/m^2^)	25.4 (21.4; 30.5)	26.7 (23.3; 31.4)	24.1 (20.4; 30.7)	0.310
Spine BMD (g/cm^2^)	1.127 (1.027; 1.243)	1.105 (1.030; 1.188)	1.100 (0.922; 1.252)	0.684
ΔSpine BMD (%)	0	−3.12 (−4.61; −1.35)^a^	2.39 (0.40; 5.22)^a,b^	<0.001
Proximal femur BMD (g/cm^2^)	0.933 (0.872; 1.045)	0.938 (0.874; 1.024)	0.939 (0.850; 1.038)	0.986
ΔProximal femur BMD (%)	0	−1.92 (−3.41; 0.64)	0.80 (−1.35; 2.65)^b^	<0.001
Femur neck BMD (g/cm^2^)	0.905 (0.837; 1.001)	0.874 (0.822; 0.976)	0.902 (0.835; 0.992)	0.841
ΔFemur neck BMD (%)	0	−2.01 (−4.54; 1.14)	1.00 (−2.15; 3.00)^a,b^	<0.001
Glucose (mmol/L)	5.30 (4.95; 5.60)	5.50 (5.10; 5.90)	5.20 (4.90; 5.55)	0.236
Phosphate (mmol/L)	1.28 ± 0.13	1.25 ± 0.12	1.12 ± 0.13^a,b^	<0.001
Calcium (mmol/L)	2.47 ± 0.09	2.46 ± 0.07	2.38 ± 0.10^a,b^	<0.001
PTH (pmol/L)	2.22 (1.83; 3.17)	2.94 (1.78; 4.46)	3.08 (2.25; 3.62)	0.056
25(OH)D (nmol/L)	64.9 ± 20.3	64.7 ± 19.9	77.1 ± 18.3^a^	0.029
β-CTX (μg/L)	0.592 (0.530; 0.864)	0.621 (0.531; 0.764)	0.248 (0.166; 0.358)^a,b^	<0.001
Δβ-CTX (%)	0	−8.5 (−23.6; 23.3)	−66.1 (−73.7; −47.5)^a,b^	<0.001
P1NP (μg/L)	80.1 (62.1; 104.1)	86.1 (61.7; 107.7)	32.9 (21.4; 51.2)^a,b^	<0.001
Δ P1NP (%)	0	0.1 (−15.6; 35.9)	−62.4 (−73.0; −48.5)^a,b^	<0.001
Osteocalcin (μg/L)	27.7 (22.0; 32.6)	30.1 (25.0; 34.4)	19.5 (17.6; 23.4)^a,b^	<0.001
B-ALP (μg/L)	13.0 (10.5; 18.6)	16.4 (13.3; 18.1)	9.5 (6.8; 11.6)^a,b^	<0.001
Sclerostin (pmol/L)	115.6 (96.9; 133.6)	103.7 (89.6; 118.0)	90.0 (74.5; 133.9)	0.032
ΔSclerostin (%)	0	−6.08 (−11.14; 4.44)	−25.19 (−32.80; −10.79)^a,b^	<0.001
TNF-R (µg/L)	2.17 ± 0.73	1.86 ± 0.73	1.74 ± 0.58^a^	0.026
ΔTNF-R (%)	0	−10.6 (−22.3; 7.4)	−28.2 (−43.2; 9.7)^a^	<0.001
IL-6R (µg/L)	41.4 ± 10.2	39.9 ± 9.5	38.4 ± 9.4	0.444
ΔIL-6R (%)	0	−1.09 (−7.93; 6.11)	−8.57 (−18.68; −3.45)^a,b^	<0.001
Estradiol (pmol/L)	19.6 (18.4; 36.1)	18.4 (18.4; 51.0)	218.5 (175.8; 268.5)^a,b^	<0.001
FSH (IU/L)	82.1 (70.1; 100.2)	85.0 (60.3; 97.3)	49.8 (32.8; 63.9)^a,b^	<0.001

Data are mean (SD) or median (IQR). Kruskal–Wallis one-way analysis of variance on ranks. No significant changes were found for hsCRP, creatinine, GMT, and TSH (data not shown). The full dataset is in the **Supplementary Table 1**.

a vs. 0.

b vs. untreated.

**Figure 3:**
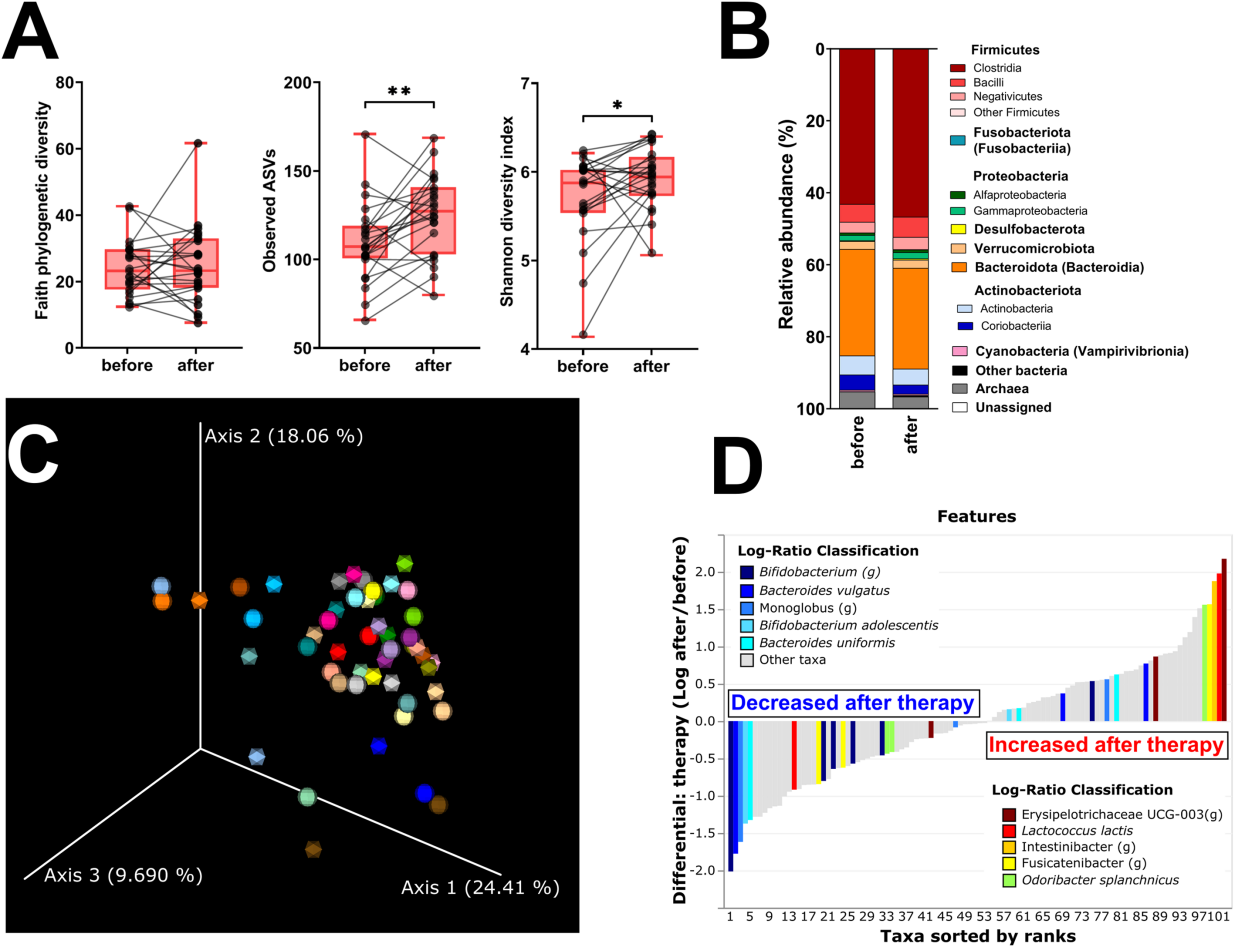
Hormonal treatment increases bacterial α-diversity and induces small but measurable shifts in β-diversity. **(A)** Changes in α-diversity after 12 months of hormonal therapy were characterized by the metrics Faith’s phylogenetic diversity, observed features, and Shannon diversity index. **(B)** β-Diversity comparison at the class level. **(C)** Principal coordinate analysis (PCoA) plot using the weighted UniFrac distance metric shows the fecal microbiota compositional differences induced by hormonal therapy. The time point before therapy is shown as a sphere and the time point after 12 months of hormonal therapy as an icosahedron. The same color represents the same patient. **(D)** Differential rankings of taxa associated with the patient’s status related to surgery with ranks estimated from multinomial regression with the top 5 species/genera related to each state highlighted. For α-diversity analyses, rarefaction was performed at 2,020 reads, and patients with lower yields in one time point were excluded from the analysis. Groups were compared with the Wilcoxon matched-pairs signed rank test or paired *t*-test **(A)**. *p < 0.05; **p < 0.01. Comparison of all clinical data for these patients is found in **Table 2**.

Faith’s PD and Simpson’s and Pielou’s evenness indices were not significantly changed, while the other metrics of α-diversity (observed ASV and Shannon diversity index) were significantly increased (**Figure 3A**; **Supplementary Figure 1C**). The changes in microbial abundances were ranked with Songbird (42), testing the effect of therapy with BMI, GMT, vitamin D, diagnosis, and age included as random effects. Although these variables led to the best model, its predictive accuracy on cross-validation samples was quite low (pseudo *Q* ^2^= 0.105333). There is a small increase in the class Clostridia (**Figure 3B**), but the overall β-diversity did not differ between the groups (**Figure 3C**). *Erysipelotrichaceae UCG-003* sp. is the most strongly associated genus with therapy, while *Bifidobacterium* sp. was the genus least associated with therapy (**Figure 3D**). Neither of these changes is, however, statistically significant.

### 3.3 Women treated with hormonal therapy have similar gut microbiota dynamics as untreated women

**Figure 4:**
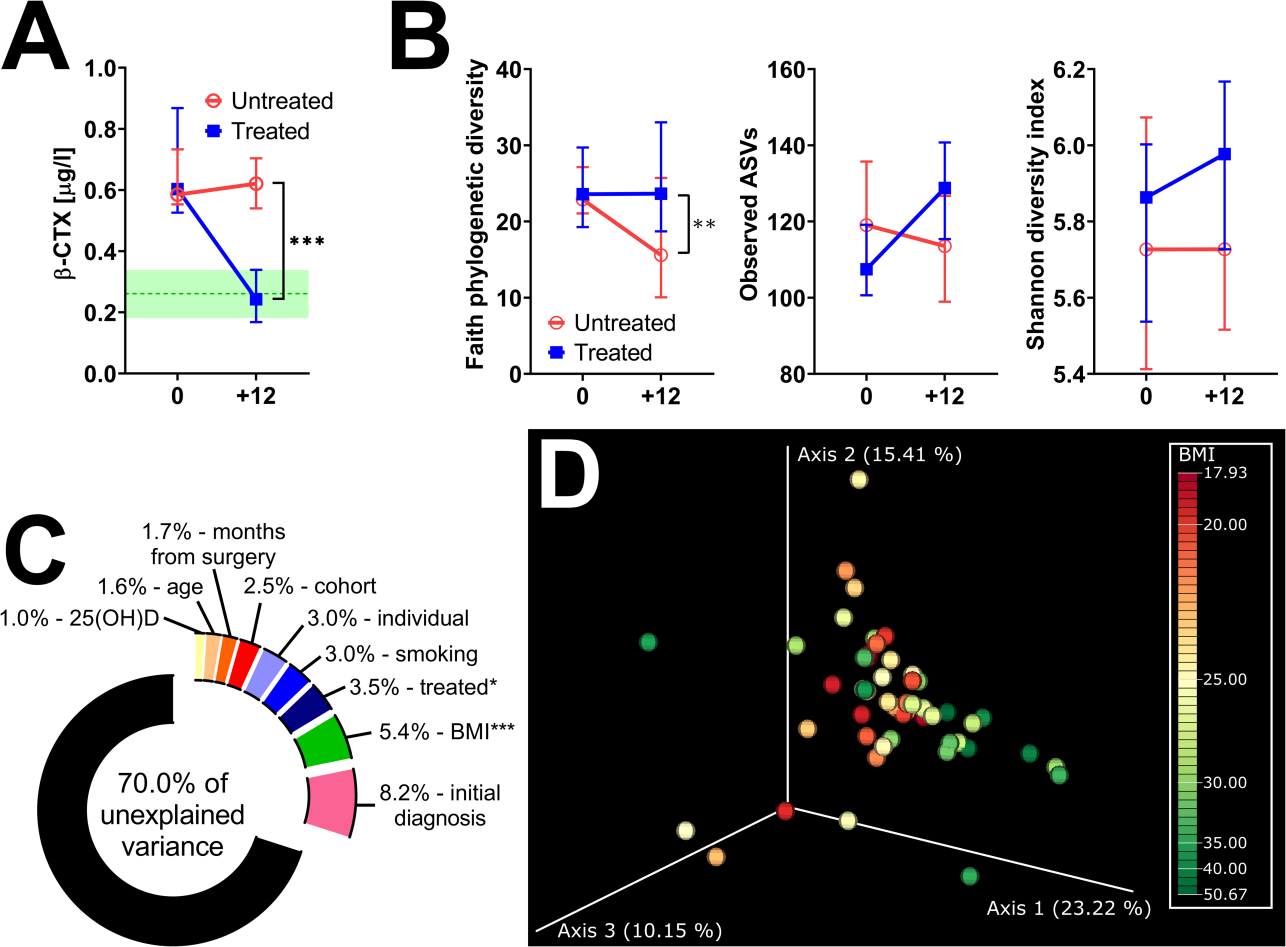
Twelve months of hormonal therapy normalized bone resorption and prevented the decrease in bacterial α-diversity. **(A)** Serum β-CTX in women after oophorectomy was normalized after 12 months of hormonal therapy (*n* = 24) but remained high in untreated (*n* = 27) women. Green line = median in women before surgery, green area = 95% CI. **(B)** Comparison of changes in α-diversity after 12 months of hormonal therapy or without it, as characterized by Faith’s phylogenetic diversity, observed features, or Shannon diversity index and analyzed by two-way ANOVA with Sidak’s multiple comparisons test. **(C)** Factors explaining microbiota variance analyzed by permutational multivariate analysis of variance using weighted UniFrac distance matrices at time 0. **(D)** Principal coordinate analysis (PCoA) plot using the weighted UniFrac distance metric shows a small shift in the fecal microbiota composition related to BMI. For α-diversity analyses, rarefaction was performed at 2,020 reads, and patients with lower yields in one time point were excluded from the analysis. *p < 0.05; **p < 0.01; ***p < 0.001.

After 12 months post-surgery, Faith’s PD continued to decrease in untreated women, while it remained stable in treated women (**Figure 4A**). Other metrics of α-diversity were not significantly different between the groups, although the observed ASVs in treated patients significantly increased during the observed period (*p* = 0.002) (**Figure 4B**; **Supplementary Figure 1D**). While there were no significant differences in β-diversity between treated and untreated patients, we tested how much microbiota variance (weighted UniFrac metrics) is explained by the nine variables (**Figure 4C**). The effect of treatment and BMI were the only factors significantly associated with shifts in β-diversity. Nevertheless, the former explained only 3.5%, while the latter explained 5.4% of the observed variation. A minor shift in axis 1 correlates with increased BMI (**Figure 4D**). There were no vegans or strict vegetarians in the observed cohort, but we identified one patient on a ketogenic diet and one with a plant-based diet with low consumption of both poultry and red meat and higher consumption of fish (pescetarian). There were no clear shifts in β-diversity during the 12 months of observation, and the gut microbiota composition was quite similar in these two women (**Supplementary Figure 2**).

### 3.4 Gut microbiota dynamics due to the introduction of sex hormones or the loss of their endogenous production

**Figure 5:**
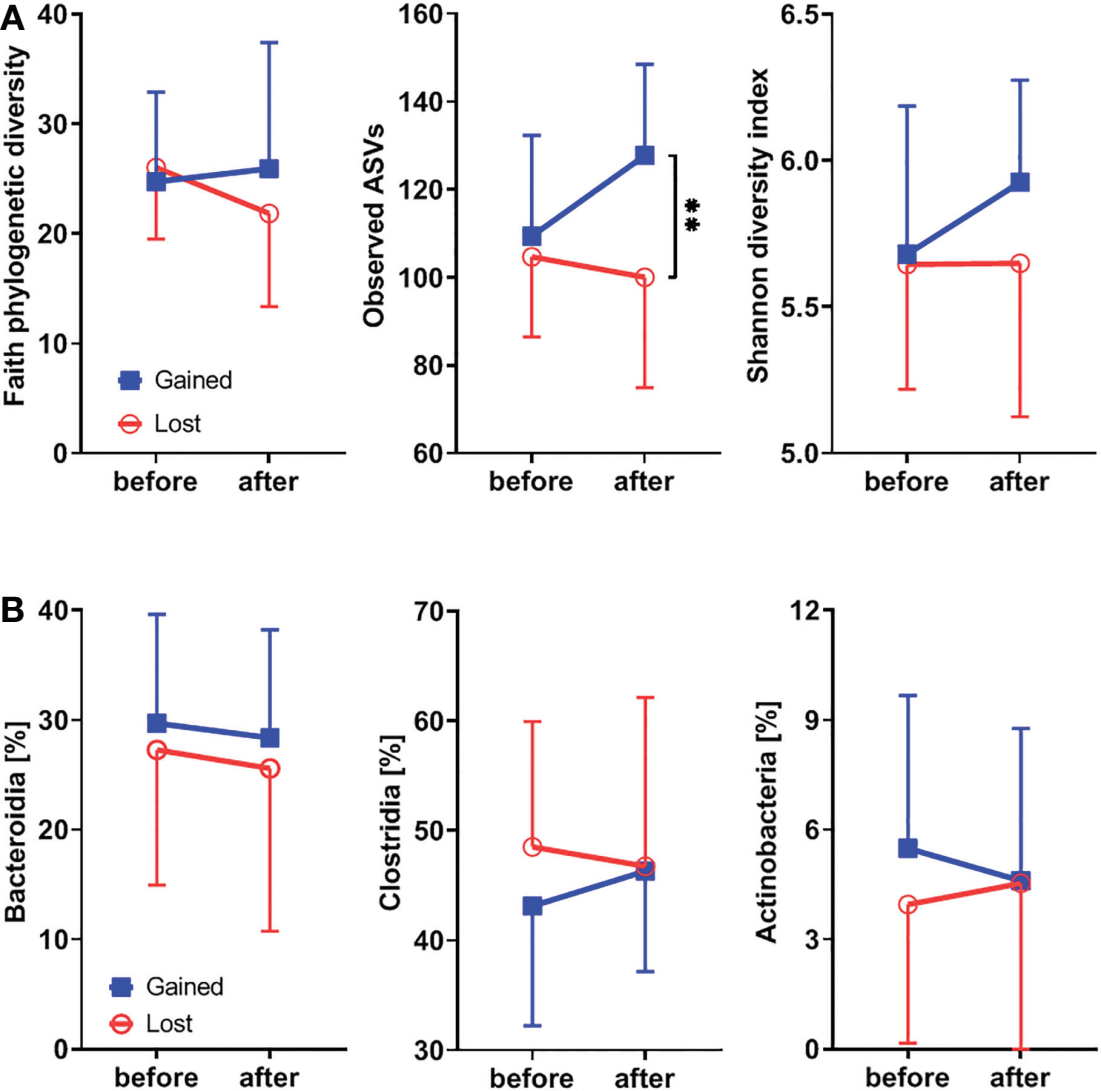
Hormonal gains lead to increased α-diversity and Clostridia abundance, while classes Bacteroidia and Actinobacteria are decreasing. **(A)** Comparison of changes in α-diversity characterized by Faith’s phylogenetic diversity, observed features, or Shannon diversity index and analyzed by two-way ANOVA with Sidak’s multiple comparisons test in patients who lost natural hormones due to surgery (lost) with those who gained hormones due to hormonal therapy (gained). In the lost group, before = before surgery and after = 12 months after the surgery. In the gained group, before = before the hormonal therapy started and after = 12 months on hormonal therapy. **(B)** Comparison of changes in selected bacterial classes based on their relative abundance using the same time points as in α-diversity analysis. For α-diversity analyses, rarefaction was performed at 2,020 reads, and patients with lower yields in one time point were excluded from the analysis. **p < 0.01.

To study the effect of hormonal levels on gut microbiota, we compared the shifts in the gut microbiota composition in patients losing their endogenous hormones (before surgery and 12 months after surgery) with those caused by their gain (before therapy and after 12 months on therapy). There were no clear differences in α-diversity (**Figure 5A**; **Supplementary Figure 1E**) or major microbial classes (**Figures 4B**, **5B**) except for the observed ASV metric of α-diversity, which increased after hormonal treatment.

### 3.5 Microbiota is not a suitable predictive factor for the state of bone metabolism

Since we could not find any major differences in gut microbiota composition, we tested the possibility of using this relative gut microbiota stability as a biomarker predicting bone resorption 6 months after surgery or 12 months after the beginning of therapy. We separated the patients into high risk (fast increase after surgery or slow decrease after therapy) and low risk based on their median Δβ-CTX and compared their microbiota at baseline. There were no distinct microbial patterns that could be used as biomarkers.

## 4 Discussion

In this prospective study, there were no significant changes in gut microbiota composition 6 months after oophorectomy, despite major changes in hormonal levels, BMD, and markers of bone remodeling and low-grade inflammation. A similar finding was reported by a study comparing α-diversity of pre- and postmenopausal women (30), suggesting that the menopause-related hormonal shift may not impact gut microbial diversity. A similar conclusion was previously drawn from a preclinical study with ovariectomized rodents (44), while others found clear shifts in the microbial profile (45). Although these animal experiments represent a relevant model of oophorectomy in humans, differences between species (e.g., mice, rats, and humans) and gut microbiota variability in human populations may markedly change the ability of the microbes to respond. Thus, only a small subgroup of patients or only animals with specific colonization in a particular animal facility may be susceptible to hormonal changes. In our study, the observed differences in β-diversity were likely due to interindividual variability rather than oophorectomy, which produced only small and generally random shifts in gut microbiota composition. While not statistically significant, some microbes are less (e.g., *L. lactis*, *A. muciniphila*) or more (e.g., genera *Monoglobus*, *Escherichia/Shigella*, and *Faecalibacterium*) abundant after surgery, thus pointing toward potential microbial targets of hormonal changes. Similarly, a tendency toward a higher abundance of *A. muciniphila* in the gut of people with higher BMD has been recently suggested by one pilot study (46). Interestingly, the bacterial communities associated with decreased BMD (i.e., osteopenia and osteoporosis) are distinct from those found in healthy postmenopausal women. However, there is no clear consensus on which specific bacterial taxa are changed (47, 48), suggesting that the affected microbes may depend on the specific cohort.

Studies on the effect of the gut microbiota on BMD have also yielded mixed results. Reduced BMD in osteopenia and osteoporosis is associated with an altered microbiota, with *Actinomyces* spp., *Eggerthella* spp., and *Lactobacillus* spp. being more common in those with osteoporosis (49). In a cross-sectional study, 35 women and 9 men over 60 years of age with osteoporosis had higher absolute and relative abundances of Bacteroidetes than 32 women and 32 men in the control group (50). In 361 postmenopausal women, the abundance of *Roseburia intestinalis* positively correlated with BMD T-score (51). In elderly subjects, Firmicutes and Actinobacteria correlated positively with BMD and T-score, while Bacteroidetes showed a negative correlation (52, 53). In older men, four bacterial genera (*Anaerofilum*, *Methanomassiliicoccus*, *Ruminiclostridium* 9, and *Tyzzerella*) were associated with improved BMD, bone structure, and strength, but the associations were not very strong (54).

In recent years, the microbiota has been implicated in the regulation of bone metabolism, and several probiotic bacteria were used to protect ovariectomized mice or postmenopausal women from bone loss (55). This effect on bone health was described in experimental studies using *Lactobacillus paracasei*, *L. acidophilus*, *L. casei*, and *Bifidobacterium longum* in ovariectomized animals (56–58). However, the effects of probiotics on bone tissue may be dependent on the systemic conditions of the host (55). *Akkermansia muciniphila* negatively correlates with obesity and other health disorders (45), and even pasteurized *A. muciniphila* protects from fat mass gain but not from bone loss (59). *Lactococcus lactis* increases fecal IgA levels and prevents the growth of H_2_S-producing enteric bacteria in a mouse model of senile osteoporosis (60). And while we found that *L. lactis* decreases after surgery and increases after hormonal therapy, these changes were not accompanied by inverse changes in H_2_S-producing Desulfobacterota or Archaea.

Probiotic interventions may improve the gut microbiome and maintain bone health. Several prospective randomized controlled trials have found several probiotic Bacilli (e.g., a mixture of *L. paracasei* DSM 13434, *Lactobacillus plantarum* DSM 15312, and *L. plantarum* DSM 15313; monotherapy with *Lactobacillus reuteri* ATCCPTA 6475 or *Bacillus subtilis* C-3102) that significantly improved bone health in postmenopausal women (61–64). However, the potential benefits of early initiated hormone therapy for the prevention of postmenopausal osteoporosis through modulating the gut microbiome have not been investigated.

We found similar hormonal and bone response profiles after surgery as in women after menopause, with a significant drop in estradiol and BMD together with an increase in the levels of markers of bone remodeling and low-grade inflammation. Bone resorption (β-CTX) reached its maximum change during the first 6 months after surgery, while BMD gradually decreased over time. Since the gut microbiota changes may not be clearly visible as early as 6 months after surgery, we followed a smaller group of patients who did not receive hormonal treatment for an additional 6 and 12 months. β-Diversity was not significantly different from the situation before surgery in either of the three time points. Interestingly, while most metrics of α-diversity did not change over time, there was a significant decrease in Faith’s PD 18 months after surgery. This discrepancy among α-diversity indices is interesting, as it suggests that 18 months after surgery the gut microbiota community became taxonomically more related while being similarly rich. This would decrease the metrics that take into account phylogenetic differences between species without affecting the other metrics. However, this correlation may also be a consequence of unrelated factors such as the sample size and short follow-up period.

As an infection prophylaxis during surgery, all patients received a short treatment with ampicillin. While antibiotics immediately impact gut microbiota, their effect wanes with time as gut microbiota recovers within 6 months even after a potent combination of multiple antibiotics (65). We did not find any significant effect on gut microbiota composition 6 months after the surgery, suggesting that ampicillin did not affect the paired comparison, although some shifts in specific microbes and their resistome may persist (66). These effects may be too subtle for us to recognize with 16S rRNA-based metataxonomic approach or the treatment regimen was too gentle and too distant to leave any major changes 6 months after surgery. This is in agreement with the majority of studies on ampicillin, as most found that gut microbiota returned to normal within 2–4 weeks after treatment (67).

Next, we followed women after surgery during 12 months of hormonal therapy to assess how this treatment influenced gut microbiota. During this time, even low hormonal doses used in our patients significantly increased serum estradiol levels and BMD and decreased serum FSH, indices of low-grade inflammation, and biomarkers of bone remodeling. However, serum estradiol and FSH levels did not reach premenopausal levels. Doses reaching follicular to periovulatory estrogen levels are needed to suppress the immune system after menopause (68, 69). During this time, Faith’s PD and both evenness metrics were unchanged, but hormonal therapy increased the observed ASVs and Shannon diversity index. Thus, unlike the changes induced by oophorectomy, therapy-induced shifts in α-diversity include an increase in the abundance of closely related species without any major shifts in higher taxonomic categories.

The differences in β-diversity were again more strongly driven by interindividual variability than by therapy. This stresses the importance of paired sample analysis as it can filter out many of these interindividual differences. In this case, the addition of BMI, serum GMT, vitamin D levels, diagnosis, and age as random effects improved the statistical model, suggesting that these factors are associated with changes in gut microbiota composition regardless of the hormonal therapy. Indeed, obesity (70) and elevated GMT (71) are both well-known modifiers of gut microbiota, and vitamin D may influence gut microbiota as well. Its supplementation significantly increases gut microbial diversity, the *Bacteroidetes*/*Firmicutes* ratio, and the abundance of *Akkermansia* and *Bifidobacterium* in healthy vitamin D-deficient women (72). In older men, serum levels of activated vitamin D (1,25(OH)_2_ vitamin D) explain variance in both α-diversity and β-diversity, as high serum 1,25(OH)_2_ vitamin D is associated with butyrate-producing bacteria in the gut (73).

Even with the correction for random effects, the predictive accuracy of the Songbird model was low. Nevertheless, some microbial shifts were associated with the therapy. Hormonal treatment decreases the abundance of *Bacteroides vulgatus*, which was recently found to negatively correlate with BMD in postmenopausal women [preprint: Lin et al. (74)]. This would suggest that *B. vulgatus* responds to estrogen levels, increasing abundance during their decline and decreasing abundance when they are present. A decrease in *B. vulgatus* after therapy may be beneficial for bone health. Its ability to deconjugate bile acids (75) may have a detrimental impact on the absorption of dietary lipids and lipid-soluble vitamins. By decreasing the concentration of glycodeoxycholic acid and tauroursodeoxycholic acid, *B. vulgatus* may even disrupt the estrous cycle in mice potentiating the effects of estrogen deficiency (76). On the other hand, estrogen therapy increased the abundance of *L. lactis*, partially restoring its decrease after surgery. These changes may influence bone health as well. *Lactococcus lactis* produces the bacteriocin nisin, which markedly decreases the inflammatory infiltrate and increases the number of fibroblast-like and osteoblast cells in the periodontal complex, which in turn prevents alveolar bone loss in a mouse model of periodontitis (77). It is not clear if these effects may be achieved systematically, but *L. lactis* upregulates genes responsible for osteoblast formation and bone matrix growth and mineralization in zebrafish (78). This suggests that an increase in *B. vulgatus* and a decrease in *L. lactis* may accelerate bone health deterioration after menopause by multiple mechanisms. However, since changes in their abundance were not statistically significant, these effects may be less important despite being found in other cohorts.

To better isolate shifts in microbiota induced by hormone therapy, we compared the shifts in gut microbiota in the treated cohort (12 months of estrogen therapy) with patients that were not treated with estrogen for a similar time. We found that 12 months of treatment normalized β-CTX to premenopausal levels, while it stayed high in untreated patients. During this time, the loss of higher taxonomic categories (Faith’s PD metrics of α-diversity) stayed at a similar level in treated patients, while it continued to decrease in untreated patients. There were no significant differences in β-diversity in ANCOM or Songbird analyses, showing that these changes do not follow any pattern related to hormonal treatment. Therefore, we used nine main factors related to our cohort and tested which explain microbiota variance in weighted UniFrac dissimilarity. We used this particular β-diversity metric because it takes into account both abundance and phylogenetic distance, which previously proved important in our data. We found that the initial diagnosis had the strongest effect, but due to its random nature, this difference was not statistically significant. The strongest factor which significantly affected β-diversity was BMI. While still being quite a modest effect, explaining only 5.4% of microbiota variability, it supports our previous observation that including BMI as a random effect in statistical models improves their performance. Gut microbiota in obese individuals differs from that of lean individuals, but it became more similar after weight loss due to sleeve gastrectomy (79).

In mice, ovariectomy induces similar obesity and shift in the gut microbiota community as a high-fat diet, only with a few bacteria specific for each type of obesity (80). Nevertheless, we did not find any significant changes in BMI during 18 months of surgery or after hormonal therapy.

Our study did not aim to establish an association of gut microbiome with the probability of osteoporosis and low-impact fractures. However, to our knowledge, this is the first prospective study to compare the associations between changes in the gut microbiome, estrogen status, BMD, and biomarkers of both bone metabolism in women before and after ovariectomy as well as in ovariectomized women after estrogen treatment. The strength of our prospective study lies in using paired samples from long-term follow-up patients. The design of this study eliminates some confounding effects on these associations. Adherence to hormone therapy was confirmed by serial measurement of serum levels of estrogen, FSH, and bone biomarkers. The key variables to assess bone status employed in this study are BMD at the lumbar spine and proximal femur and biomarkers reflecting the extent of type I collagen degradation and synthesis (β-CTX and P1NP), two recommended markers of bone resorption and bone formation, respectively (81). The decrease in BMD values at the lumbar spine, proximal femur, and femur neck in our untreated women, at the 6th, 12th, and 18th postoperative months, compared with the preoperative values, is in line with previously published studies (82–86). Also, the results of our follow-up of women who underwent oophorectomy before their natural menopause are in line with previously published studies of other biomarkers of bone remodeling (87–92), sclerostin (93), and TNF-R and IL6-R (94–96).

Our study has several limitations. First, clinical diagnosis and variable hormonal status in patients indicated for oophorectomy, as well as several lifestyle factors that can influence estrogen levels after surgery, such as nutritional status and exercise, were not addressed. Questionnaires on dietary habits allowed us only to identify dietary extremes, and while patients were advised to maintain their usual physical activity and diet throughout the study, smaller changes or shorter shifts cannot be ruled out. Diet is the main factor influencing the gut microbiota composition, allowing gut microbiota to quickly adapt to dietary changes (97). However, these changes are only temporary with clear shifts after 3 months and return nearly to the original baseline 6 and 12 month after the dietary intervention (98). Thus, the resilience of gut microbiota together with the paired comparisons probably minimalized the impact of diet. Second, given the short duration of the study and the low number of patients, changes in gut microbiota should be interpreted with caution. The short-term effects of an acute drop in estrogen levels after ovariectomy should be compared with the effects of gradual hormonal changes during the menopausal transition. Third, the effects of estrogens on bone remodeling are dose-dependent. Therefore, the effects of treatment with only 1 mg of estradiol, prescribed to our patients by their gynecologists, may differ from the effects of endogenous estradiol in healthy premenopausal women. Fourth, while the 16S rRNA-based approach can precisely characterize the gut microbiota composition, it cannot identify subtle shifts in microbial metabolism or other functions.

## 5 Conclusions

Here, we provide evidence that neither loss of estrogens due to oophorectomy nor their gain due to subsequent hormonal therapy is associated with a specific gut microbiota signature. This is despite the fact that bone metabolism was markedly influenced by these changes. We found that oophorectomy leads to a small decrease in phylogenetically corrected α-diversity in the long term and hormonal therapy prevents this decrease. We found that BMI is associated with a significant shift in gut microbiota composition, and while it was quite modest, it shows the importance of controlling for personal and environmental factors, ideally by collecting paired samples over a long period of time.

## Data availability statement

The datasets presented in this study can be found in online repositories. The names of the repository/repositories and accession number(s) can be found below: PRJNA914622 (SRA).

## Ethics statement

The studies involving human participants were reviewed and approved by The Ethics Committee of the Institute of Rheumatology, Prague, Czech Republic. The patients/participants provided their written informed consent to participate in this study.

## Author contributions

Conceptualization: JS and MKv. Methodology: ZJ, NG and ZR. Software: MKo. Formal analysis: ZJ, JS, SC, MKo and MKv. Investigation: ZJ, SC, NG and ZR. Data curation: ZJ, JS, MKo and MKv. Writing—original draft: ZJ, JS, SC and MKv. Writing—review and editing: all authors. Funding acquisition: JS and MKv. All authors contributed to the article and approved the submitted version.
