# Supplementary material for: Interindividual differences contribute to variation in microbiota composition more than hormonal status: A prospective study
Source: Front Endocrinol (Lausanne). 2023 Mar 8;14:1139056. doi: 10.3389/fendo.2023.1139056 (PMC10081494; doi:10.3389/fendo.2023.1139056)
Supplement: Supplementary file 1 [file Image_1.pdf]

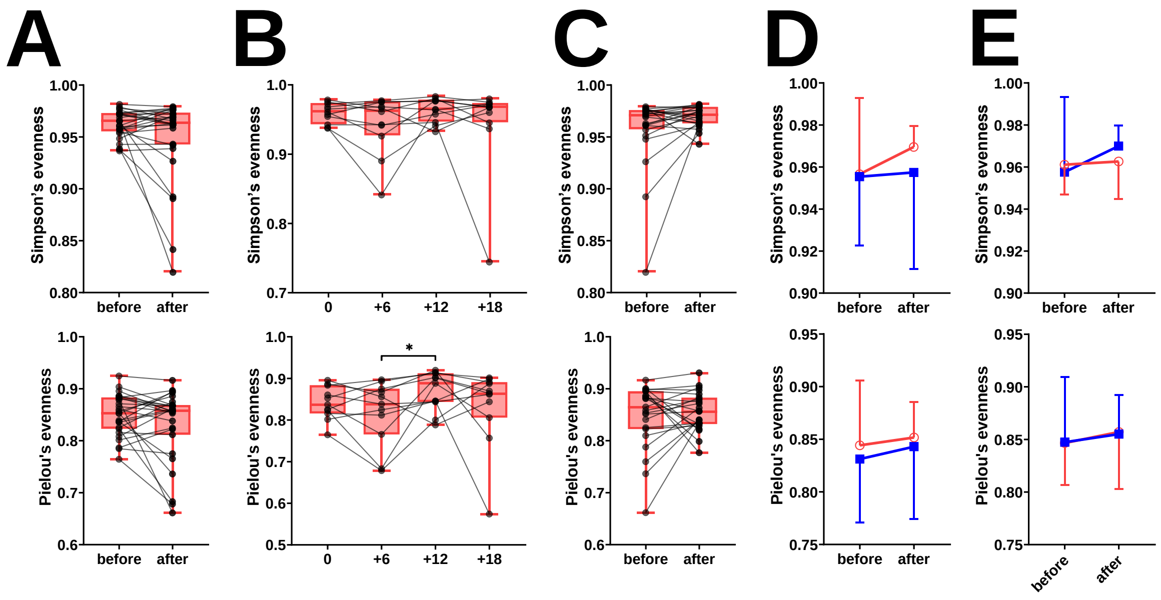

Supplementary Figure 1. Additional metrics of α-diversity comparing changes **(A)** 6 months after surgery related to Figure 2A, **(B)** 6, 12 and 18 months after surgery related to Figure 2E and **(C)** after 12 months of hormonal therapy related to Figure 3A. Comparison of changes in α-diversity between **(D)** 12 months of hormonal therapy or without it related to Figure 4B and **(E)** 12 months after hormonal loss due to the surgery and hormonal gain during therapy related to Figure 5A.
