# Supplementary material for: Interindividual differences contribute to variation in microbiota composition more than hormonal status: A prospective study
Source: Front Endocrinol (Lausanne). 2023 Mar 8;14:1139056. doi: 10.3389/fendo.2023.1139056 (PMC10081494; doi:10.3389/fendo.2023.1139056)
Supplement: Supplementary file 2 [file Image_2.pdf]

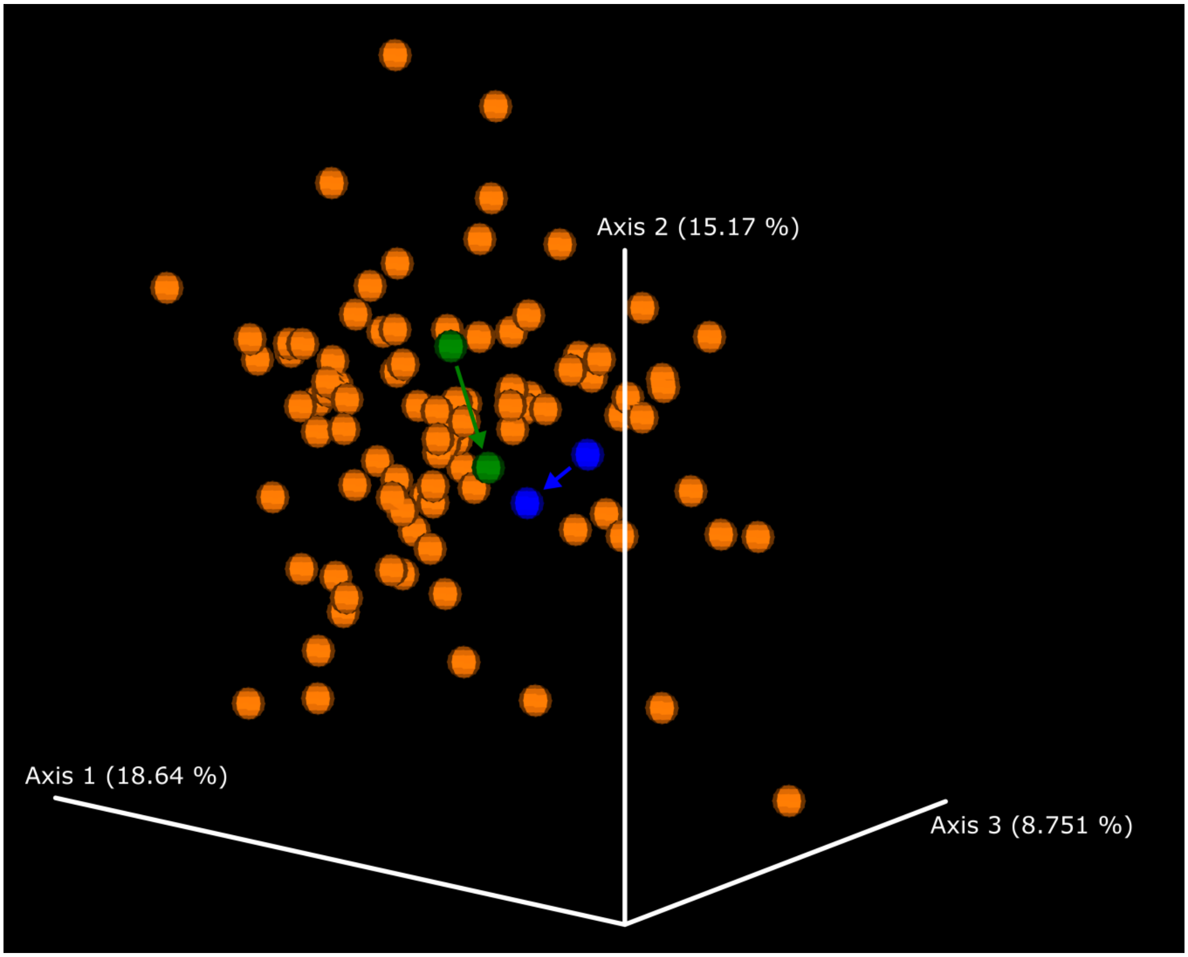

Supplementary Figure 1. There are no major shifts in gut microbiota β-diversity related to extreme diets. Principal coordinates analysis (PCoA) plot using the Weighted UniFrac distance metric shows the fecal microbiota compositional differences induced by hormonal therapy. Blue spheres shows patient on ketogenic diet and green is pescetarian, the arrow show shift during 12 month of observation.
